# A Rapid Antimicrobial Susceptibility Test for Determining *Yersinia pestis* Susceptibility to Doxycycline by RT-PCR Quantification of RNA Markers

**DOI:** 10.3389/fmicb.2019.00754

**Published:** 2019-04-16

**Authors:** Ohad Shifman, Ida Steinberger-Levy, Ronit Aloni-Grinstein, David Gur, Moshe Aftalion, Izhar Ron, Emanuelle Mamroud, Raphael Ber, Shahar Rotem

**Affiliations:** ^1^Department of Biochemistry and Molecular Genetics, Israel Institute for Biological Research, Ness Ziona, Israel; ^2^Department of Physical Chemistry, Israel Institute for Biological Research, Ness Ziona, Israel

**Keywords:** *Yersinia pestis*, doxycycline, antimicrobial susceptibility test, AST, MIC, RT-PCR, blood cultures

## Abstract

Great efforts are being made to develop new rapid antibiotic susceptibility tests to meet the demand for clinical relevance versus disease progression. This is important especially in diseases caused by bacteria such as *Yersinia pestis*, the causative agent of plague, which grows rapidly *in vivo* but relatively slow *in vitro*. This compromises the ability to use standard growth-based susceptibility tests to obtain rapid and proper antibiotic treatment guidance. Using our previously described platform of quantifying antibiotic-specific transcriptional changes, we developed a molecular test based on changes in expression levels of doxycycline response-dependent marker genes that we identified by transcriptomic analysis. This enabled us to determine the minimal inhibitory concentration of doxycycline within 7 h compared to the 24 h required by the standard CLSI test. This assay was validated with various *Y. pestis* strains. Moreover, we demonstrated the applicability of the molecular test, combined with a new rapid bacterial isolation step from blood cultures, and show its relevance as a rapid test in clinical settings.

## Introduction

The development of rapid antimicrobial susceptibility tests (ASTs) is a major challenge in light of the worldwide escalation in antibiotic resistance. Antibiotic-resistant pathogens, either naturally occurring or intentionally developed as bioterror agents, are of growing concern to public health. Hence, prompt and accurate medical care should rely on rapid methods to identify the bacteria and to determine their susceptibility to the antibiotic treatment of choice. Bacterial susceptibility is determined by standard ASTs, guided by the Clinical and Laboratory Standards Institute (CLSI) guidelines or the European Committee on Antimicrobial Susceptibility Testing (EUCAST) (Matuschek et al., 2014; Clinical and Laboratory Standards Institute [CLSI], 2015, 2018). As these tests are growth dependent, they are time consuming and may delay proper treatment, especially upon infection with a pathogenic bacteria that grows rapidly *in vivo* but slowly *in vitro.* An example of such a bacterium is *Yersinia pestis* (*Y. pestis*), the causative agent of plague, which is classified by the Centers for Disease Control and Prevention (CDC), based on its rapid disease progression, severity, and person-to-person transmission (Inglesby et al., 2000), as a Tier 1 select agent^1^. To date, the standard AST for *Y. pestis* uses the microdilution technique that requires 24 h of incubation (Clinical and Laboratory Standards Institute [CLSI], 2015), not including the time for prior bacterial isolation that requires an additional 2 days. Bearing in mind that high mortality rates are observed if antibiotic treatment is not initiated within 18 to 24 h following the onset of symptoms (Inglesby et al., 2000), rapid and reliable ASTs are needed during plague outbreaks, such as the one reported recently on the island of Madagascar^2^.

Great efforts have been made by us and others to develop new rapid bacterial isolation methods and AST approaches (Steinberger-Levy et al., 2007, 2016; Zahavy et al., 2012, 2018; Aloni-Grinstein et al., 2015, 2017; Meka-Mechenko et al., 2017; Huang et al., 2018). For a recent review, see Maugeri et al. (2018). These include improved recording of bacterial growth in the presence of the tested antibiotic, as in agar-diffusion tests such as the Etest^^®^^ (Jacobs et al., 1992; Walsh et al., 2002; Yusof et al., 2008), plate reader (Reller et al., 2009), digital time-lapse microscopy (Fredborg et al., 2015), and microscopic observation of colony formation (Price et al., 2014). Moreover, novel approaches to monitoring rapid biological changes during the exposure of bacterial culture to antibiotic treatment rather than the final output of death versus survival have also been developed. These include the measurement of membrane potential (Nuding and Zabel, 2013; Zahavy et al., 2018), label-free cytometry (Huang et al., 2018) Raman spectroscopy for biomarkers (Liu et al., 2016), and the measurement of transcriptome output (Steinberger-Levy et al., 2016; Khazaei et al., 2018). Molecular-based methods, such as resistance genes identification (Anjum et al., 2017; Fleece et al., 2018) or clonotyping (Tchesnokova et al., 2017), may also assist in antibiotic selection; however, these methods are limited in their ability to precisely identify MICs and may require prior knowledge of the clones’ sensitivity profile. Recent whole-genome sequencing technologies have also been applied for the rapid prediction of antibiotic resistance. However, a comprehensive analysis of those efforts by EUACST concluded that those approaches are still not well-developed enough to assist in clinical decision making (Ellington et al., 2017).

We have previously reported a proof of concept for a rapid molecular AST for determining *Y. pestis* susceptibility to ciprofloxacin, one of the CDC-recommended antibiotics for *Y. pestis* treatment^3^. The molecular assay is based on quantification of the alterations in the expression levels of ciprofloxacin-specific early-response *Y. pestis* genes, identified by a transcriptomic screen (Steinberger-Levy et al., 2016). To extend the applicability of our molecular approach, we established a rapid molecular AST for the determination of *Y. pestis* susceptibility to doxycycline, an additional antibiotic recommended by the CDC for post-exposure prophylaxis^3^. As different antibiotics inhibit bacterial growth through different biological mechanisms, it is expected that the bacterial transcriptome will be antibiotic dependent, although some overlap may occur. Thus, doxycycline transcriptome profiling was performed to identify potential marker genes suitable for rapid doxycycline AST. Furthermore, we challenged our molecular susceptibility assay using a modeled clinical setting of *Y. pestis*-spiked blood cultures. For this purpose, we employed an SST isolation procedure (Steinberger-Levy et al., 2007; Aloni-Grinstein et al., 2017) followed by a new rapid filtration step to separate the bacteria from the blood culture within 30 min, compared to the 48 h required for colony isolation by agar culturing. Determination of the doxycycline MIC by our molecular assay was achieved in 7 h compared to the 24 h required by the standard CLSI method. The overall time (including the bacterial isolation steps) required for MIC determination by our newly described procedure was 7.5 h versus the 72 h required by the standard CLSI test, suggesting the applicability of our test in clinical settings.

## Materials and Methods

### Bacterial Strains and Growth Conditions

The *Y. pestis* strains Kimberley53 (Kim53), EV76, A1122, and Kim53pPCP1^−^pCD1^−^ were previously reported (Ben-Gurion and Hertman, 1958; Flashner et al., 2004). The KIM D27 strain (Garcia et al., 1999) was kindly supplied by Prof. Mikael Skurnik, University of Helsinki, Finland. Non-virulent plasmid-cured Kim53pPCP1^−^pCD1^−^ isolates with reduced susceptibility to doxycycline were generated by spontaneous mutant selection according to previously described methods (Lindler et al., 2001; Udani and Levy, 2006; Louie et al., 2007a,b, 2011a,b). The isolation was performed in compliance with the Israeli law for working with selected agents and was approved by the institutional review board. Experiments conducted using virulent strains were performed using BSL-3 containment procedures. Experiments using non-virulent strains were performed using BSL-2 containment procedures.

Bacteria were routinely grown at 28°C on Difco^TM^ brain heart infusion agar (BHIA, BD Cat# 241830) or on BIN (Ber et al., 2003). For the broth-microdilution tests (see section “Standard Doxycycline Susceptibility Test”) and the molecular AST (see section “Molecular Doxycycline Susceptibility Tests”), cation-adjusted BBL^TM^ Mueller-Hinton II Broth (MHB, BD Cat# 212322) was used. For the blood culture experiments, bacteria (10^3^ CFU) were spiked in 10 ml of human blood, obtained from the National Blood Services, MDA, Israel, under MDA research permit 08-0122. The spiked blood was transferred into BACTEC^TM^ Plus Aerobic/F Culture vials (BD, Cat# 442192). The vials were agitated at 180 rpm and 37°C for 48 h. Doxycycline was purchased from Sigma-Aldrich (D9891) as a powder, and stock solutions of 16 mg/ml in distilled water were prepared and stored at −70°C until use. Each experiment included a standard microdilution AST on *Y. pestis* to verify doxycycline concentrations and activity following dilutions to assay concentrations.

### Isolation of *Y. pestis* From Blood Cultures

For bacterial isolation from blood-culture vials, 8 ml of the culture was transferred to a serum-separator tube (SST, Vacuette Cat# 455071), and the tube was centrifuged at 1700 × *g* for 15 min at 20°C to separate the red blood cells. At the end of the centrifugation, the supernatant was carefully discarded, and the bacteria, lying on the gel matrix, were suspended by gentle vortexing in 4 ml of MHB and were then transferred to a sterile 50-ml tube. The gel was washed with additional 4 ml of MHB. The resulting 8-ml suspension was filtered through a Minisart^^®^^ NML syringe filter with a 5-μm pore size (Sartorius, Cat# 17594-K) pre-equilibrated with MHB, to remove white blood cell remnants.

### Standard Doxycycline Susceptibility Test

Antimicrobial susceptibility tests were conducted by broth-microdilution tests according to the CLSI guidelines (Clinical and Laboratory Standards Institute [CLSI], 2015). An inoculum of 5 × 10^5^−1 × 10^6^ CFU/ml suspended in MHB was added at a 1:1 volume to a 96-well plate (TPP, Cat# 92696) containing duplicates of twofold serial dilutions of doxycycline in MHB at a final volume of 0.1 ml. Bacteria grown in MHB without the addition of doxycycline served as a growth control in each assay. The 96-well plate was incubated at 28°C for 24 h in an Infinite 200 plate reader (TECAN), and growth was monitored by measuring the optical density at 630 nm (OD_630_) at 1-h intervals. A 28°C incubation temperature was used because it better supported bacterial growth without affecting the MIC (Frean et al., 2003; Hernandez et al., 2003; Heine et al., 2015), which led to shorter AST durations and easier MIC determination. The MIC values were defined as the lowest doxycycline concentration that reduced growth to less than 10% of the OD_630_ measured for the growth control. No growth was verified by visual inspection. Each assay was performed in at least three independent experiments.

### Transcriptomic Screen for Doxycycline-Responsive Genes

The transcriptomic screen for doxycycline-responsive genes was conducted essentially as previously described for ciprofloxacin (Steinberger-Levy et al., 2016). BHIA-plated *Y. pestis* Kim53 bacteria were suspended in MHB to OD_660_ = 0.33 (∼1 × 10^8^ CFU/ml) as measured by a Novaspec Plus spectrophotometer (Amersham). The culture was then diluted 1:200 in MHB to obtain the standard CLSI-recommended concentration of 5 × 10^5^ to 1 × 10^6^ CFU/ml and incubated at 150 rpm and 28°C for 2 h for the adjustment of the culture to MHB. Aliquots of 100 ml of culture were added to 0.5-L Erlenmeyer flasks containing 1 ml of 100× doxycycline stock solution for a final concentration of 8 to 0.125 μg/ml at a twofold serial dilution. An untreated culture served as a control. The bacteria were then incubated with continuous shaking at 150 rpm and 28°C for 2 or 3 h. Following incubation, duplicates of 48 ml from each bacterial culture were centrifuged at 3200 × *g* for 15 min at 4°C. The supernatants were discarded, and the bacterial pellets were frozen in liquid nitrogen and stored at −70°C until RNA purification (see section “RNA Purification and Quantitative RT-PCR Assays”).

The RNA was used for DNA microarray transcriptomic analysis as previously described for identifying ciprofloxacin-responsive genes (Steinberger-Levy et al., 2016). DNA microarray analysis was performed using our custom Agilent 8 × 15K slide containing probes representing the *Y. pestis* CO92 genome. Each sample was labeled with Cy3-CTP or Cy5-CTP, while the untreated control was inversely labeled. Statistical analysis was performed using the Limma (Linear Models for Microarray Data) package from the Bioconductor project using the “read.maimages” functions. Background subtraction and LOWESS normalization were performed for each array. The Benjamini-Hochberg false discovery rate was used to correct for multiple comparisons. The transcriptomic-derived fold change (FC) was calculated for each gene as the median FC value calculated for the 3 to 5 probes representing the gene.

### Molecular Doxycycline Susceptibility Tests

Molecular doxycycline susceptibility tests were conducted on bacterial suspensions originating from agar plates or blood culture vials. For bacteria grown on agar plates, the bacteria were suspended in MHB to OD_660_ = 0.33 (∼1 × 10^8^ CFU/ml). Bacteria isolated from blood cultures (see section “Isolation of *Y. pestis* From Blood Cultures”) were quantified by a Petroff-Hausser counting chamber under a light microscope. The bacteria were then brought to the standard CLSI-recommended inoculum concentration of 5 × 10^5^ CFU/ml in MHB to serve as the inoculum for the molecular ASTs.

The inoculum was incubated with continuous shaking at 200 rpm and 28°C for 2 h to reset the bacterial transcriptome. Aliquots were then added (1:1 in volume) to serial twofold dilutions of doxycycline in a total volume of 0.5 ml in a 24-well plate (Costar, Cat# 3524). The 24-well plate was incubated for 2 h at 28°C. The entire-well volumes were transferred to 2-ml tubes (Sarstedt, Cat# 72.693.005) and centrifuged at 5000 × *g* for 5 min at 4°C. At the end of the centrifugation, the supernatants were discarded, and RNA was extracted and purified from the bacterial pellets, as described in Section “RNA Purification and Quantitative RT-PCR Assays”. Quantitative reverse-transcriptase PCR (qRT-PCR) for the *mgtB* and *bioD* markers (see section “RNA Purification and Quantitative RT-PCR Assays”) was conducted on the purified RNA. Normalized FCs were calculated for each doxycycline-treated sample, as described in Section “Fold Change Calculations”. The molecular MIC (mMIC) was defined as the doxycycline concentration exhibiting the maximal normalized FC.

### RNA Purification and Quantitative RT-PCR Assays

Bacterial pellets were suspended in 80 μl of lysozyme solution (0.4 mg/ml in TE, Sigma-Aldrich Cat# L6876 and T9285 for the lysozyme powder and the 100× TE buffer solution, respectively) and incubated for 5 min at room temperature. The RNA was then purified using the RNeasy RNA mini kit (QIAGEN, Cat# 74104) according to the manufacturer’s instructions but using 270 μl of the RLT buffer. Residual DNA was removed by 15 min on-column digestion using RNase-free DNase (QIAGEN, Cat# 79254).

The qRT-PCRs were conducted as 25-μl single-tube reactions. Each reaction contained 0.5 M KCl, 0.1 M Tris-HCl, pH 8.8, 1 μM SuperROX^^®^^ (Biosearch Technologies, Cat# SR-1000-10), 3 mM MgCl_2_, 0.2 mM dNTPs, 0.6 μM from each primer, 0.3 μM TaqMan^^®^^ probe, 0.25 μl of Sensiscript^^®^^ RT (QIAGEN, Cat# 205213), 1 U of MyTaq^TM^ HS DNA polymerase (Bioline, Cat# BIO-21111) and 5 μl of the purified RNA. Primers and probes were ordered from Integrated DNA Technologies, Inc. qRT-PCRs were performed in an Applied Biosystems 7500 real-time PCR system under the following conditions: 50°C for 30 min, 95°C for 3 min, 40 cycles of 94°C for 15 s and 60°C for 35 s. Cycle threshold (Ct) values were extracted using the 7500 real-time PCR system Sequence Detection Software (version 1.4).

### Fold Change Calculations

The FC in expression levels of a marker gene in a doxycycline-exposed sample compared to its expression levels in an unexposed control was calculated from the marker’s Ct of the exposed culture (Ct_exposed_) and the Ct of the unexposed culture (Ct_unexposed_) using the equation FC = 2^−ΔCt^, where ΔCt is Ct_exposed_-Ct_unexposed_.

The FC ratio between the two marker genes, *mgtB* (FC_mgtB_) and *bioD* (FC_bioD_), termed *mgtB/bioD* FC, was calculated for each doxycycline concentration (DC) using the equation *mgtB/bioD* FC_DC_ = FC_mgtB_/FC_bioD_.

The normalized FC for each DC, defined as the difference in the *mgtB/bioD* FC values between twofold-increased adjacent concentrations (DC and 2 × DC) and normalized by DC, was calculated as: normalized FC = (*mgtB/bioD* FC_2 × DC_-*mgtB*/*bioD* FC_DC_)/DC.

## Results

Previously (Steinberger-Levy et al., 2016), we described a rapid molecular AST based on the qRT-PCR quantitation of the changes in the expression of mRNA markers upon a short 2-h exposure of *Y. pestis* to ciprofloxacin and showed that this test can predict the standard MIC. In the present study, we extend the applicability of our molecular AST approach by searching for doxycycline-responsive mRNA markers that can rapidly determine *Y. pestis* susceptibility to doxycycline.

### Identification of *Y. pestis* Doxycycline-Responsive mRNA Markers

**FIGURE 1:**
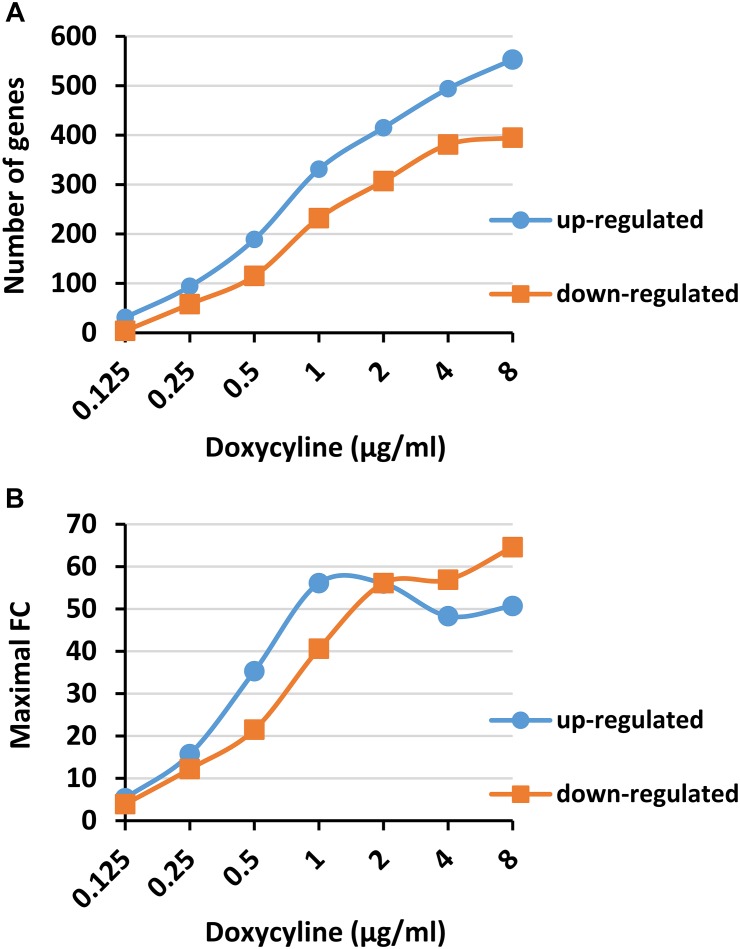
Dose-dependent expression of *Yersinia pestis* genes upon exposure to doxycycline. *Y. pestis* Kim53 bacteria were exposed for 2 h to the indicated concentrations of doxycycline. After 2 h, the bacteria were harvested and subjected to transcriptomic analysis using a custom Agilent DNA microarray. **(A)** The number of genes that were upregulated (blue circle) or downregulated (orange square) by at least threefold upon exposure to doxycycline. **(B)** The maximal change in expression levels that was observed at each doxycycline concentration in the upregulated (blue circle) or downregulated genes (orange square).

To search for mRNA transcripts that may be suitable as markers for the rapid detection of *Y. pestis* susceptibility to doxycycline, we conducted a transcriptomic analysis with the fully virulent *Y. pestis* Kim53 strain in response to a short exposure (2 or 3 h) to doxycycline under CLSI-standard conditions. The chosen doxycycline concentration range included a subinhibitory concentration (0.125 μg/ml), the standard MIC range of *Y. pestis* (0.25 to 1 μg/ml) and higher concentrations up to the susceptibility breakpoint concentrations (2 to 8 μg/ml). The changes in the gene expression levels in the exposed bacteria were compared to their basal levels in an unexposed control bacterial culture. Transcriptomic analysis was conducted with the Agilent custom-tailored DNA microarray that was used and verified in our previous study (Steinberger-Levy et al., 2016). This DNA microarray was designed to contain *Y. pestis* CO92 chromosomal and plasmidial genes based on its published and annotated full genome (Parkhill et al., 2001). Both Kim53 and CO92 belong to the biovar Orientalis and exhibit high sequence similarity (Tidhar et al., 2009). The transcriptomic analysis of the 2-h doxycycline-exposed bacteria revealed that the numbers of genes that were upregulated (Figure 1A, blue circle) or downregulated (orange square) were dose-dependent, involving up to several hundred genes at higher doxycycline concentrations. Furthermore, a substantial number of genes (94 and 58 up- and downregulated genes, respectively) changed more than threefold at a 1× MIC concentration (0.25 μg/ml, as determined in a parallel standard microdilution assay). Likewise, the maximal FC in expression levels obtained for any of the upregulated or downregulated genes increased in a dose-dependent manner (Figure 1B), reaching 50- and 60-fold at the highest doxycycline concentrations. To further refine our search and retrieve the most suitable mRNA markers, we looked for candidate genes that, in addition to a substantial change in their expression levels compared to an unexposed control culture (of at least 3 FC), possessed a substantial change in expression levels (of at least 2.5 FC) over bacterial culture exposed to sub-MIC concentrations of 0.5× MIC (0.125 μg/ml, column 1× MIC/0.5× MIC in Table 1). In addition, to avoid a “bell-shaped” response, wherein high doxycycline concentrations result in reduced gene expression, we searched for genes that retained high response levels (above 5 FC) at the highest tested doxycycline concentrations (column 32× MIC/0.5× MIC in Table 1). Applying these criteria of 3 FC above unexposed culture, 2.5 FC above 0.5× MIC and 5 FC at 32× MIC above 0.5× MIC resulted in 15 candidate genes (Table 1), of which 4 were upregulated and 11 were downregulated. Applying the same criteria to the transcriptomic analysis of bacteria exposed to doxycycline for 3 h revealed similar results (Supplementary Table S1). We observed more genes (9 upregulated and 18 downregulated), but most of the genes from the 2-h screen were also present in the analysis of the 3-h exposure. Thus, the 2-h exposure time was chosen for the development of the rapid molecular AST.

**Table 1:** The change in the candidate gene expression after 2 h of exposure to doxycycline: transcriptomic results.

				Fold change^a^					
			
				Doxycycline (μg/ml)					
			
	0.125	0.25	0.5	1	2	4	8	MIC FC ratio	
Gene name (ORF)^b^	0.5× MIC	1× MIC^c^	2× MIC	4× MIC	8× MIC	16× MIC	32× MIC	$\frac{1\times \text{MIC}}{0.5\times \text{MIC}}$	$\frac{32\times \text{MIC}}{0.5\times \text{MIC}}$
*lcrF* (YPCD1.49)	2.2	7.2	14.6	28.8	29.7	23.0	27.5	3.2	12.3
*yscA* (YPCD1.50)	4.3	15.7	35.3	56.1	56.0	41.2	29.2	3.6	6.8
*ORFC* (YPCD1.65)	2.0	5.1	6.9	12.4	14.5	12.5	10.3	2.6	5.2
*mgtB* (YPO1661)	3.5	10.5	19.6	36.2	44.8	48.3	47.9	3.0	13.8
*iucA* (YPO0989)	−1.4	−3.6	−8.6	−15.3	−17.4	−13.0	−14.5	2.5	10.2
*bioD* (YPO1154)	−1.3	−3.4	−7.2	−9.7	−10.5	−11.5	−12.4	2.6	9.4
*fyuA* (YPO1906)	−2.3	−7.2	−14.7	−22.6	−24.6	−29.7	−26.4	3.2	11.5
*irp5* (YPO1907)	−2.9	−9.2	−14.0	−20.5	−23.5	−30.0	−30.1	3.2	10.3
*irp4* (YPO1908)	−3.3	−11.9	−18.5	−35.1	−39.9	−39.5	−45.8	3.6	13.9
*irp3* (YPO1909)	−3.9	−12.2	−18.2	−27.0	−31.6	−34.0	−36.4	3.1	9.3
*irp1* (YPO1910)	−3.7	−12.0	−16.0	−25.5	−28.0	−28.8	−35.1	3.3	9.6
*irp2* (YPO1911)	−2.8	−11.2	−21.5	−37.4	−41.2	−55.1	−56.2	4.0	20.0
*irp6* (YPO1913)	−2.9	−9.6	−19.7	−35.4	−44.9	−55.4	−55.8	3.4	19.5
*irp7* (YPO1914)	−3.0	−10.8	−21.0	−40.6	−56.1	−56.9	−64.6	3.6	21.7
*irp9* (YPO1916)	−2.0	−7.6	−15.6	−21.9	−26.5	−30.7	−31.4	3.7	15.4

*^a^The change in the gene expression levels was determined for the Y. pestis Kim53 strain exposed for 2 h to the indicated doxycycline concentrations versus the expression in unexposed control bacteria. Negative FCs represent downregulated responses. The red and green intensity gradients are according to the FC values. ^b^Gene names and ORFs are based on the annotation of Y. pestis CO92 chromosome and plasmids sequences (Accession Nos. AL590842, AL117211, AL117189, and AE017046). ^c^The 1× MIC value (0.25 μg/ml doxycycline) was determined in a parallel standard microdilution assay.*

Notably, most of the candidate genes belong to common biological pathways (Table 2), underscoring the significance of the transcriptomic screen. These biological pathways are different from the biological pathways that we observed in the search for the ciprofloxacin-responsive genes. Interestingly, while most of the doxycycline-responsive pathways were involved in nutrient synthesis and transport, suggesting a nutrient-deprivation stress, ciprofloxacin treatment induced mainly DNA repair and SOS response pathways (Steinberger-Levy et al., 2016), probably due to the difference in the mode of actions of these two antibiotics.

**Table 2:** Biological functions of the candidate genes.

Genes	Biological function^a^
*lcrF, yscA*, *ORFC*	pCD1 plasmid-derived virulence factors (Plano and Straley, 1995; Schwiesow et al., 2015)
*mgtB*	Mg^+2^ transporting ATPase (Ford et al., 2014)
*iucA*	Iron ion homeostasis (Forman et al., 2007)
*bioD*	Biotin biosynthetic process
*fyuA, irp1, irp2, irp3, irp4, irp5, irp6, irp7, irp9*	Iron uptake system, *pgm* locus (Iteman et al., 1993; deAlmeida et al., 1994; Buchrieser et al., 1999; Gaddy et al., 2014)

*^a^Biological functions were obtained from the Gene Ontology Consortium (www.geneontology.org), from the annotation of the Y. pestis CO92 sequence and the aforementioned references.*

### qRT-PCR Quantification of the Doxycycline-Responsive mRNA Markers

**FIGURE 2:**
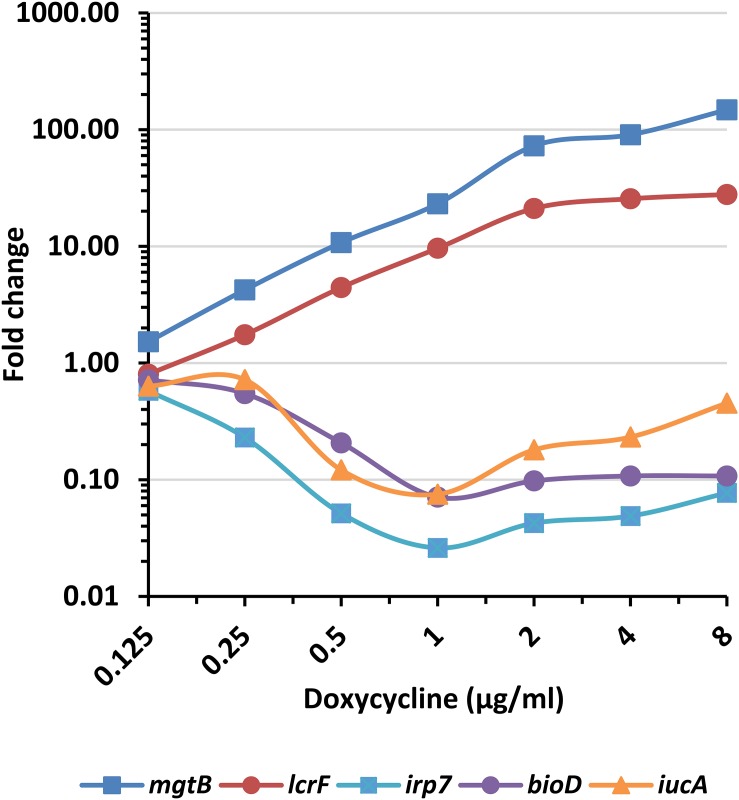
Expression pattern of the doxycycline-responsive genes. *Y. pestis* Kim53 bacteria were exposed for 2 h to the indicated concentrations of doxycycline. The bacterial cultures were then harvested, RNA was extracted and the FCs in the expression levels of the indicated genes, compared to their levels in an unexposed bacterial culture, were determined by qRT-PCR. *mgtB* and *lcrF* are upregulated genes, while *irp7*, *bioD*, and *iucA* are downregulated.

To verify our transcriptomic results and to establish an RT-PCR assay for the quantification of the marker genes, we developed TaqMan-based qRT-PCR assays for two upregulated (*mgtB* and *lcrF*) and three downregulated (*irp7*, *bioD*, and *iucA*) genes (see Supplementary Table S2 for the primer and probe sequences). The genes were selected according to the following criteria: representation of different biological pathways (Table 2), the ratio of FC at 1× MIC over 0.5× MIC (Table 1) and the ability to find an efficient qRT-PCR assay for the genes. The qRT-PCR analysis was performed using similar settings as in the transcriptomic screen but at smaller volumes, and it confirmed that *mgtB* and *lcrF* are upregulated in response to doxycycline treatment while *irp7*, *bioD*, and *iucA* are downregulated, all in a dose-dependent manner (Figure 2).

The FC in the expression levels of the marker genes are usually normalized using housekeeping genes by the ΔΔCt approach (Livak and Schmittgen, 2001). We instead opt to use the downregulated genes. This enabled us to achieve higher FCs by taking into account the cumulative change in expression of both genes in response to doxycycline exposure, while each gene serves as an internal normalization control for RNA integrity. Preliminary results showed that this approach is valid and that the FC ratio of *mgtB* to *bioD* (*mgtB/bioD* FC) had the best predictive power for the molecular assay and was therefore chosen for the mMIC determination.

### Determination of the Molecular MIC

**FIGURE 3:**
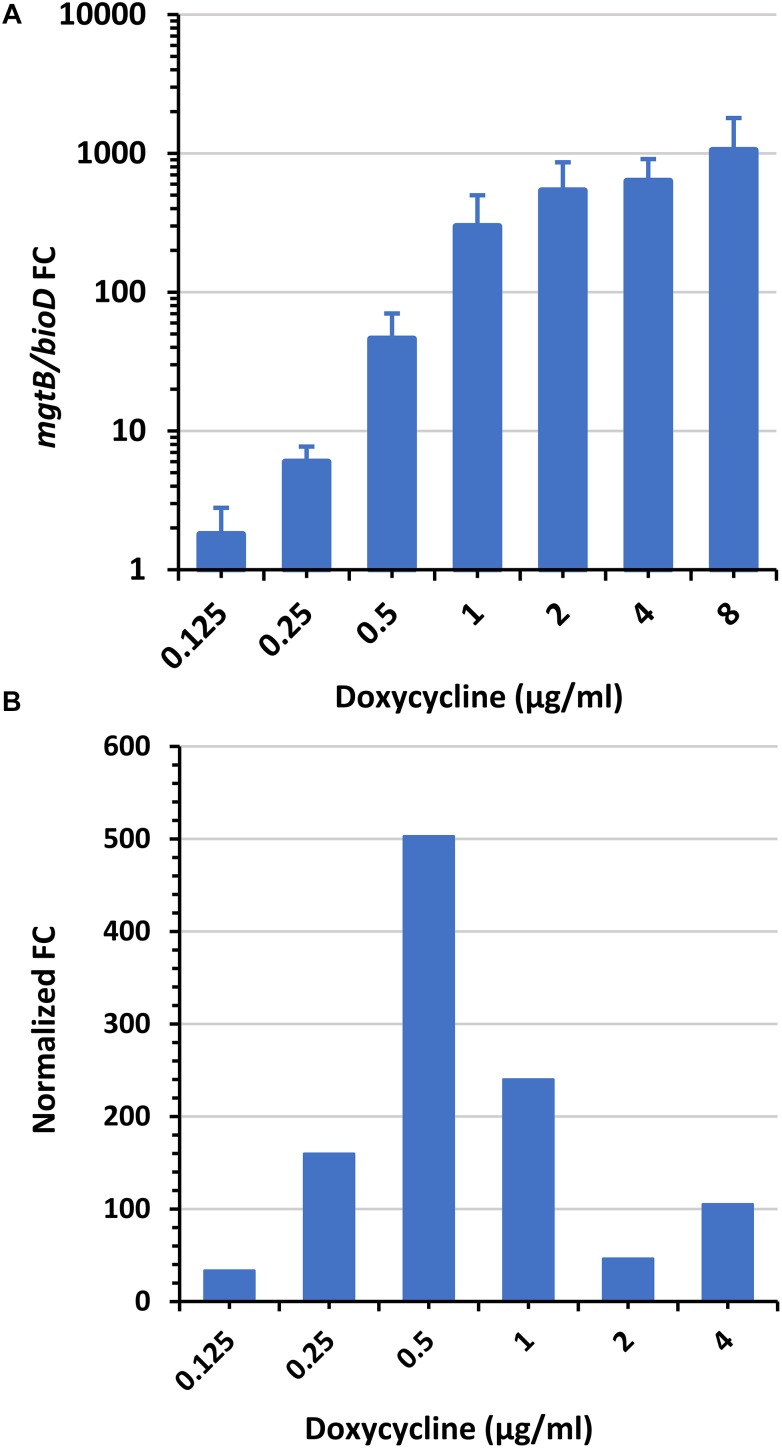
*mgtB/bioD* FC and normalized FC values as a function of doxycycline concentration. *Y. pestis* Kim53 culture was exposed to the indicated concentrations of doxycycline. After 2 h, the bacterial cultures were harvested, RNA was extracted and the expression levels of *mgtB* and *bioD* (by means of Ct values) were determined using qRT-PCR. **(A)** *mgtB/bioD* FC values were calculated for each doxycycline concentration from the Ct values. Bars and error bars are averages and their standard deviations from three independent experiments. **(B)** The normalized FCs are presented for each doxycycline concentration. The mMIC was determined as the doxycycline concentration with the maximal normalized FC (0.5 μg/ml). The standard MIC of the bacteria is 0.25–1 μg/ml.

To determine the mMIC, *Y. pestis* cultures were exposed for 2 h to a wide range of doxycycline concentrations. qRT-PCR assays for the *mgtB* and *bioD* genes were conducted on the RNA extracts, and the *mgtB/bioD* FC value for each doxycycline concentration was determined as described in Section “Fold Change Calculations”. As shown in Figure 3A, exposure of *Y. pestis* Kim53 to increasing doxycycline concentrations resulted in an increase in the *mgtB/bioD* FC values while maintaining a high FC at higher doxycycline concentrations. This doxycycline-mediated response was also observed in additional *Y. pestis* strains EV76 and A1122, which belong to the biovar Orientalis, and KIM D27, which belongs to the biovar Mediaevalis (Supplementary Figure S1). We found that the highest *mgtB/bioD* FC increment, when normalized to the corresponding doxycycline concentration (normalized FC, see section “Fold Change Calculations” for mathematical calculation), coincided with the standard MIC. Hence, we applied this formula to determine the mMIC. Figure 3B depicts the normalized FC values obtained for the Kim53 strain. The maximal normalized FC value was obtained at 0.5 μg/ml, which is within the range of the MIC values (0.25–1 μg/ml) found for this strain by the standard CLSI AST. Likewise, similar analyses conducted on the other *Y. pestis* strains (Table 3) showed that in all cases, the mMIC values were within the standard MIC range obtained for these strains.

**Table 3:** Molecular MIC analysis of *Y. pestis* strains.

	Normalized FC^a^			
Doxycycline (μg/ml)	Kim53^b^	EV76^b^	KIM D27^b^	A1122^b^
0.125	33 ± 10	30 ± 6.3	6.1 ± 4.0	18 ± 5.0
0.25	160 ± 93	130 ± 21	45 ± 25	42 ± 4.8
0.5	**503 ± 355**^c^	**433 ± 86^c^**	**173 ± 94**^c^	152 ± 13
1	240 ± 170	296 ± 65	126 ± 78	**257 ± 116**^c^
2	46 ± 30	128 ± 173	−8.3 ± 14	0.7 ± 53
4	105 ± 119	−20 ± 54	−6.5 ± 11	34 ± 55

*^a^The presented results are the average and standard deviation of three independent experiments. ^b^The standard doxycycline MIC of the strains is 0.25–1 μg/ml. ^c^The mMIC of the strains is indicated in bold.*

These results indicate that the mMIC assay can be used to predict the MIC values of a wide range of *Y. pestis* strains. Notably, the mMIC results were obtained after a significantly shorter exposure (2 h) to the antibiotics than the standard AST incubation time (24 h).

### Determination of the mMIC of *Y. pestis* Derivatives With Reduced Susceptibility to Doxycycline

Next, we challenged our molecular AST using strains with reduced susceptibility to doxycycline. To that end, we isolated bacteria with reduced doxycycline susceptibility from the non-virulent strain Kim53pPCP1^−^pCD1^−^, which lacks the pPCP1 and the pCD1 plasmids, which are important for the manifestation of virulence (Flashner et al., 2004; Perry and Bearden, 2008) but do not affect the MIC value compared to the virulent strain Kim53 (Table 4). The isolates with reduced susceptibility were generated through the selection of spontaneous mutants by consecutive rounds of plating on increasing amounts of doxycycline. This selection process resulted in isolates with increased MIC values (Table 4) compared to the standard MIC of the parental strain (0.25–1 μg/ml). Notably, the strains represented the three susceptibility categories – susceptible (S), intermediate (I), and resistant (R).

**Table 4:** Standard MIC values of the avirulent *Y. pestis* Kim53pCD1^−^pPCP1^−^ isolates with reduced doxycycline susceptibility.

*Y. pestis* isolates	Doxycycline MIC (μg/ml)	Susceptibility category^a^
Kim53	0.25–1	S
Kim53pCD1^−^pPCP1^−^ (parental)	0.25–1	S
#36	2	S
#36-4	8	I
#36-4-18	32	R

*^a^Doxycycline susceptibility breakpoint categories (S-susceptible; ≤4 μg/ml, I-intermediate; =8 μg/ml and R-resistant; ≥16 μg/ml) were derived from (Clinical and Laboratory Standards Institute [CLSI], 2015).*

These isolates were tested with our molecular AST, and the mMIC obtained was similar to the standard MIC as determined for each isolate, i.e., identical or within one twofold dilution (Table 5).

**Table 5:** Molecular MIC analysis of avirulent *Y. pestis* Kim53pCD1^−^pPCP1^−^ isolates with reduced doxycycline susceptibility.

	Normalized FC^a^			
Doxycycline (μg/ml)	Parental^b^	#36^b^	#36-4^b^	#36-4-18^b^
0.125	70 ± 64	1.2 ± 0.8	2.8 ± 0.3	1.9 ± 2.2
0.25	117 ± 58	10 ± 3.6	−0.1 ± 0.1	0.1 ± 0.4
0.5	**350 ± 19^c^**	40 ± 24	2.5 ± 0.3	−0.2 ± 0.1
1	275 ± 158	107 ± 56	7.5 ± 3.4	0.0 ± 0.4
2	20 ± 50	**172 ± 88**^c^	27 ± 19	0.7 ± 0.5
4	11 ± 14	73 ± 50	**47 ± 9.8^c^**	1.2 ± 0.2
8	1.9 ± 14	4.7 ± 27	28 ± 9.2	4.4 ± 0.9
16	9.4 ± 11	8.5 ± 8.3	24 ± 16	12 ± 1.4
32	6.5 ± 3.6	4.3 ± 2.7	10 ± 8.4	**20 ± 24**^c^

*^a^The presented results are the average and standard deviation of three independent experiments. ^b^The standard doxycycline MIC values in parallel experiments were 0.5, 2.0, 8.0, and 32 μg/ml for the parental, #36, #36-4, and #36-4-18 isolates, respectively. ^c^The mMIC of the isolates is indicated in bold.*

These results demonstrate that our rapid test has the potential to accurately predict the MIC values of both wild-type doxycycline-sensitive *Y. pestis* strains as well as strains with reduced susceptibility to doxycycline. This observation is of great importance in light of naturally occurring (Welch et al., 2007; Cabanel et al., 2018) and potential willfully generated resistant *Y. pestis* isolates.

### Application of the Rapid Molecular AST for MIC Determination of *Y. pestis* Grown in Blood Cultures

**FIGURE 4:**
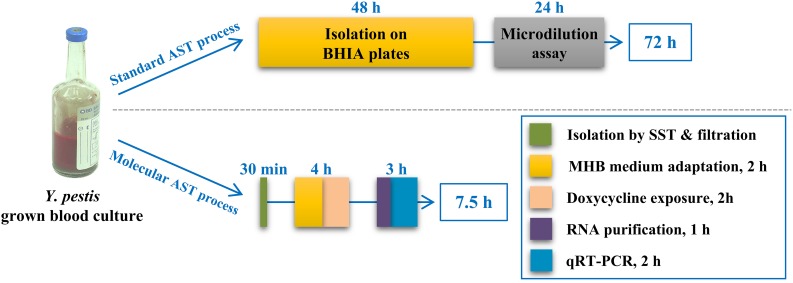
Schematic presentation of the standard and molecular ASTs for the determination of *Y. pestis* susceptibility to doxycycline. The scheme presents the steps of the standard CLSI-based and the molecular susceptibility tests for the determination of the doxycycline susceptibility of *Y. pestis* originating from blood cultures. The duration of each step is indicated, as well as the overall test duration.

Blood cultures are a major enrichment source for clinical bacterial isolation. Thus, we applied our molecular AST to blood culture samples. To that end, we spiked blood samples with *Y. pestis* EV76 to an initial concentration of 10^2^ CFU/ml. The spiked blood was transferred into BACTEC^TM^ Plus Aerobic/F Culture vials and incubated at 37°C to allow for bacterial growth. These conditions were chosen to simulate the setup used in the clinics for bacterial enrichment from blood samples. After 48 h, the bacterial culture was harvested, and the sample was subjected to a rapid (30-min) isolation step. We first used an SST-based fractionation step (Steinberger-Levy et al., 2007; Aloni-Grinstein et al., 2017), as described in Section “Isolation of *Y. pestis* From Blood Cultures,” to separate the bacteria from the red blood cells. Next, the bacteria suspended in MHB were filtered through a 5-μm syringe filter to remove the white blood cells and cell debris. We then quantified the bacteria in the filtrate using a Petroff-Hausser counting chamber under a light microscope and subjected the bacteria to a 2-h recovery period at 28°C with agitation at 200 rpm prior to performing the molecular AST. This recovery step was added to allow the bacterial transcriptome to adjust from the blood culture conditions (e.g., 37°C, blood culture media) to the conditions used for the mAST (e.g., 28°C, MHB). The bacterial culture was then exposed for 2 h to a wide range of doxycycline concentrations and subjected to the molecular AST. As shown in Table 6, the mMIC was 0.5 μg/ml, which was in agreement with the standard CLSI MIC. Notably, the overall time required for MIC determination by our newly described procedure was 7.5 h versus the 72 h required by the standard CLSI test (Figure 4).

**Table 6:** Molecular MIC analysis of *Y. pestis* EV76 isolated from blood cultures.

Doxycycline (μg/ml)	Normalized FC^a^
0.125	45 ± 9.3
0.25	93 ± 4.4
0.5	**189 ± 40**^b^
1	51 ± 30
2	27 ± 22
4	7.8 ± 7.6

*^a^The presented results are the average and standard deviation of three independent experiments. ^b^The mMIC of the isolates is indicated in bold. The doxycycline MIC values in standard parallel experiments were also 0.5 μg/ml.*

These results demonstrate the great potential of our molecular AST, in combination with rapid bacterial isolation steps, to be used as a tool for the rapid determination of antibiotic susceptibility of bacteria that are slow-growing in clinical settings.

## Discussion

The development of rapid ASTs is of great importance, especially for prompt medical care of patients with rapidly progressing life-threatening bacterial disease. The classic ASTs are based on the comparative growth of isolated bacteria in the presence of the tested antibiotic. However, some of these bacteria grow slowly under *in vitro* conditions. The discrepancy between their rapid *in vivo* replication and their slow *in vitro* multiplication rate may render AST results clinically irrelevant. *Y. pestis*, the causative agent of plague, belongs to this category of pathogenic bacteria. In recent decades, we and others (Lindler et al., 2001; Ber et al., 2003; Steinberger-Levy et al., 2007, 2016; Bugrysheva et al., 2016; Aloni-Grinstein et al., 2017; Meka-Mechenko et al., 2017; Zahavy et al., 2018) have developed strategies to overcome this obstacle for *Y. pestis* by addressing the time-consuming isolation step and by developing short alternative AST approaches, such as a rapid molecular AST for the determination of *Y. pestis* susceptibility to ciprofloxacin (Steinberger-Levy et al., 2016). This molecular AST is based on monitoring the expression levels of specific mRNA markers, which are rapidly altered in response to ciprofloxacin exposure at levels to which the tested isolate is susceptible, thus allowing for the determination of the MIC of isolated bacterial colonies within 7 h rather than the 24 h required for the standard CLSI microdilution assay. In this study, we used a similar approach to establish a rapid molecular-based AST for the determination of *Y. pestis* susceptibility to doxycycline. We identified several candidate mRNA markers that were substantially altered in response to a short exposure (2 h) to doxycycline. As expected, the genes that responded to doxycycline exposure were different from those found in the ciprofloxacin screen, which highlights the specificity of the selected mRNA markers in the molecular AST based on the tested antibiotic for a defined bacterium. Although ciprofloxacin-responsive genes are mainly involved in stress-response pathways (Steinberger-Levy et al., 2016), most doxycycline-responsive genes are related to nutrient deprivation responses. This difference in gene response likely reflects the difference in the mechanisms of action of the two antibiotics. Doxycycline is a bacteriostatic protein synthesis inhibitor that may stimulate nutrient deprivation signals, while ciprofloxacin is bactericidal inhibiting DNA helicase, leading to double-strand DNA breaks and the stalling of replication, thus inducing DNA repair and SOS response genes. Investigating the expression of the doxycycline- and ciprofloxacin-responsive genes in *Y. pestis* under exposure to additional antibiotics and their expression in additional pathogens might help elucidate both distinctive and general physiological pathways and mechanisms involved in these responses. Advances in transcriptome analysis, such as RNA-seq, and the uncovering of mutual marker genes, both to different pathogens or various antibiotics, may contribute to a broader application of our mAST.

Validation of the doxycycline-responsive genes using qRT-PCR confirmed that the genes found are indeed up- or downregulated and enabled us to quantify the change in their expression levels in an accurate and rapid manner. Applying sophisticated quantification and normalization methods, where the change in the gene expression of the upregulated gene, *mgtB*, was determined in comparison to the change in the downregulated gene, *bioD*, resulted in both an increase in the FC obtained for the individual genes and served as internal normalization gene instead of a housekeeping gene, reducing sample-to-sample variation due to the difference in RNA extraction yields. We confirmed that the *mgtB/bioD* FC is universally induced in a dose-dependent manner in the various *Y. pestis* strains tested in this study, as well as in *Y. pestis* isolates with reduced susceptibility to doxycycline. In our previous study, we used a global threshold to determine the mMIC value. The limitation of this approach is the requirement of extensive validation to set the threshold value. In this study, we employed intrinsic criteria based on the finding that the *mgtB/bioD* FC change per doxycycline concentration (normalized FC) is bell shaped with a maxima at the MIC concentration. The bell-shaped curves observed for the normalized FC results from the expression profile of the selected marker genes, which present the highest change in response to doxycycline at the MIC concentration. This phenomenon might suggest that approaching the MIC concentration, the bacteria rapidly respond to the stress induced by the antibiotics, thus resulting in enhanced and repressed transcription of these genes. At higher concentrations, although gene expression still remains high, the change in expression levels is reduced (Figure 3A), resulting in the reduction of the normalized FC values, which takes into account the doxycycline concentration.

Overall, using this assay, 96% of the molecular doxycycline ASTs we conducted (*n* = 27) were within one twofold dilution of the standard MIC, fulfilling the requirement for a newly developed AST of >95% agreement within one double dilution of the standard MIC (Jorgensen and Ferraro, 2009) and FDA documentation^4^.

Because of the great potential of our molecular AST with respect to the time course in clinical settings, we examined its applicability to clinically relevant samples, namely, blood cultures. The protocol included a simple and rapid bacterial isolation step requiring 30 min followed by the described molecular AST, yielding an MIC determination within 7.5 h, compared to the 3 days required for isolation and susceptibility testing by the standard CLSI method. This can allow faster accurate medical treatment, reducing morbidity and mortality rates.

## Author Contributions

OS, IS-L, RA-G, DG, EM, RB, and SR contributed to the design of the study and data interpretation. OS, IS-L, RA-G, DG, MA, and RB contributed to the experiments and data acquisition. OS, IR, and SR contributed to mathematical and statistical analyses. OS, IS-L, RA-G, EM, RB, and SR contributed to the writing of the manuscript.

## Conflict of Interest Statement

The authors declare that the research was conducted in the absence of any commercial or financial relationships that could be construed as a potential conflict of interest.

## Footnotes

1 https://www.selectagents.gov/selectagentsandtoxinslist.html

2 https://afro.who.int/health-topics/plague/plague-outbreak-situation-reports

3 https://www.cdc.gov/plague/healthcare/clinicians.html

4 https://www.fda.gov/MedicalDevices/DeviceRegulationandGuidance/GuidanceDocuments/ucm080564.htm
