# Supplementary material for: A Rapid Antimicrobial Susceptibility Test for Determining Yersinia pestis Susceptibility to Doxycycline by RT-PCR Quantification of RNA Markers
Source: Front Microbiol. 2019 Apr 16;10:754. doi: 10.3389/fmicb.2019.00754 (PMC6477067; doi:10.3389/fmicb.2019.00754)
Supplement: Supplementary file 2 [file Table_1.docx]

**Supplementary Table S1: The change in the candidate gene expression after 3 h of exposure to doxycycline: transcriptomic results.**

|  | **Fold Change^a^** | | | | | | |  | |
| --- | --- | --- | --- | --- | --- | --- | --- | --- | --- |
|  | **doxycycline (µg/ml)** | | | | | | |  | |
|  | **0.125** | **0.25** | **0.5** | **1** | **2** | **4** | **8** |  | |
| **Gene Name^b^** | **0.5xMIC** | **1xMIC^c^** | **2xMIC** | **4xMIC** | **8xMIC** | **16xMIC** | **32xMIC** | $\frac{\mathbf{1xMIC}}{\mathbf{0.5xMIC}}$ | $\frac{\mathbf{32xMIC}}{\mathbf{0.5xMIC}}$ |
| *YPCD1.22c* | 1.6 | 4.5 | 9.8 | 19.2 | 23.5 | 20.4 | 23.5 | 2.8 | 14.7 |
| *virG* | 2.3 | 6.7 | 19.3 | 23.9 | 21.2 | 16.9 | 15.7 | 2.9 | 6.7 |
| *lcrF** | 2.0 | 9.9 | 36.1 | 48.5 | 42.9 | 34.9 | 31.4 | 5.0 | 15.7 |
| *yscA** | 2.5 | 13.4 | 47.6 | 59.8 | 39.6 | 30.7 | 23.0 | 5.3 | 9.1 |
| *yscB* | 1.9 | 9.1 | 21.7 | 22.7 | 16.4 | 13.7 | 9.9 | 4.7 | 5.1 |
| *YPO1649* | 1.9 | 5.4 | 10.5 | 10.9 | 11.6 | 11.4 | 12.1 | 2.8 | 6.3 |
| *mgtC* | 1.7 | 5.2 | 13.0 | 17.3 | 18.1 | 18.0 | 18.5 | 3.0 | 10.7 |
| *mgtB** | 2.9 | 11.8 | 27.7 | 39.5 | 43.2 | 43.0 | 39.6 | 4.0 | 13.5 |
| *iucA** | -1.1 | -3.1 | -15.0 | -18.1 | -15.3 | -15.2 | -12.6 | 2.9 | 11.7 |
| *iucB* | -1.2 | -3.2 | -9.2 | -8.9 | -12.3 | -11.0 | -12.4 | 2.6 | 10.1 |
| *iucC* | -1.3 | -3.5 | -6.5 | -10.4 | -12.2 | -13.6 | -14.1 | 2.8 | 11.3 |
| *iucD* | -1.4 | -4.3 | -10.6 | -12.8 | -14.0 | -15.3 | -13.7 | 3.0 | 9.5 |
| *bioA* | -1.3 | -3.6 | -9.8 | -10.0 | -10.6 | -8.9 | -12.1 | 2.9 | 9.6 |
| *bioC* | -1.5 | -3.6 | -8.7 | -9.4 | -11.4 | -11.5 | -12.9 | 2.5 | 8.9 |
| *bioF* | -1.3 | -3.6 | -13.9 | -14.4 | -15.7 | -15.9 | -16.0 | 2.7 | 12.2 |
| *fyuA** | -2.2 | -7.8 | -17.7 | -20.7 | -24.3 | -27.1 | -27.7 | 3.5 | 12.5 |
| *irp5** | -2.4 | -8.4 | -12.9 | -16.1 | -21.9 | -23.6 | -25.5 | 3.5 | 10.6 |
| *irp4** | -2.5 | -11.0 | -20.1 | -31.6 | -33.3 | -36.6 | -45.0 | 4.4 | 17.9 |
| *irp3** | -2.9 | -11.4 | -17.5 | -22.8 | -29.1 | -34.4 | -34.2 | 3.9 | 11.7 |
| *irp1** | -2.9 | -9.6 | -14.8 | -23.0 | -23.4 | -27.8 | -29.5 | 3.3 | 10.2 |
| *irp2** | -2.5 | -9.5 | -20.8 | -26.9 | -39.7 | -53.1 | -52.4 | 3.8 | 20.7 |
| *irp6** | -2.3 | -10.8 | -31.9 | -41.1 | -49.8 | -51.8 | -44.0 | 4.7 | 19.1 |
| *irp7** | -2.8 | -12.2 | -33.2 | -44.0 | -57.5 | -67.0 | -64.4 | 4.3 | 22.8 |
| *irp8* | -2.2 | -6.7 | -14.7 | -19.2 | -24.9 | -25.0 | -28.2 | 3.0 | 12.6 |
| *irp9** | -2.0 | -9.2 | -24.1 | -25.1 | -28.5 | -31.8 | -36.5 | 4.6 | 18.3 |

^a^ The change in the gene expression levels was determined for *Y. pestis* Kim53 strain exposed for 3 h to the indicated doxycycline concentrations versus the expression in unexposed control bacteria. Negative FCs represent downregulated responses. The red and green intensity gradients are according to the FC values.

^b^ Gene names are based on the annotation of *Y. pestis* CO92 chromosome and plasmid sequences (accession numbers: AL590842, AL117211, AL117189, AE017046)

^c^ The 1xMIC (0.25 µg/ml doxycycline) was determined in a parallel standard microdilution assay.

^*^ Genes that were also found in the 2-hour exposure screen.
