# Supplementary material for: A Rapid Antimicrobial Susceptibility Test for Determining Yersinia pestis Susceptibility to Doxycycline by RT-PCR Quantification of RNA Markers
Source: Front Microbiol. 2019 Apr 16;10:754. doi: 10.3389/fmicb.2019.00754 (PMC6477067; doi:10.3389/fmicb.2019.00754)
Supplement: Supplementary file 3 [file Table_2.DOCX]

**Supplementary table S2: Primers and Probes used in this study**

| Gene |  | Sequences |  |
| --- | --- | --- | --- |
| *mgtB* | Forward primer | GCGGGAAGGTGATGACATCT | |
|  | Reverse primer | CCAGAGCTTGTAGCGTTGCA | |
|  | Probe | 6FAM-TCCACTGGATGCTGCCCGCC-BHQ1 | |
| *lcrF* | Forward primer | TGCGTCGTGGCAGCTATG | |
|  | Reverse primer | CCGGGTAATGGAATCCAAAGT | |
|  | Probe | 6FAM-TCGATGTGGTACAAAAGAACCCTGCCA-BHQ1 | |
| *irp7* | Forward primer | GGTAGTGACGGGCACTCTGAA | |
|  | Reverse primer | CTGCGAAGCGCATAATCATC | |
|  | Probe | 6FAM-TCGCCTTTTTGATTGCCGCCG-BHQ1 | |
| *bioD* | Forward primer | CGCCATTGCTGGGAATTATT | |
|  | Reverse primer | TCAAATCCAAGTAGTGCCCAAGA | |
|  | Probe | 6FAM-AAATCCGGTGACAACACGCCGTG-BHQ1 | |
| *iucA* | Forward primer | AAGGTAATTTCTTCTGCTATCTACACGAT | |
|  | Reverse primer | GGTGCAGAGGGTTAACGAAATC | |
|  | Probe | 6FAM-AAACACCATCGTTGATCCGGCAGTG-BHQ1 | |
